# Supplementary material for: Benchmarking of tools for axon length measurement in individually-labeled projection neurons
Source: PLoS Comput Biol. 2021 Dec 8;17(12):e1009051. doi: 10.1371/journal.pcbi.1009051 (PMC8824366; doi:10.1371/journal.pcbi.1009051)
Supplement: S1 Text — (DOCX) [file pcbi.1009051.s001.docx]

**SUPPORTING INFORMATION**

**Relationship between real and projected length of unidimensional structures in 2D under zero-anisotropy condition**

Let us consider a unidimensional structure of *N* joined segments of equal length *L* embedded in a two-dimensional space (Fig A left in S1 Text). For each segment $i\epsilon\left\{ 1,\ldots, N \right\}$ it holds:

$l_{i}=L \sin(\theta_{i})$,

where $l_{i}$ is the length of the horizontal projection of segment *i* (Fig A left in S1 Text) and $\theta_{i}$ is the angle between the segment and the positive vertical axis (Fig A right in S1 Text).


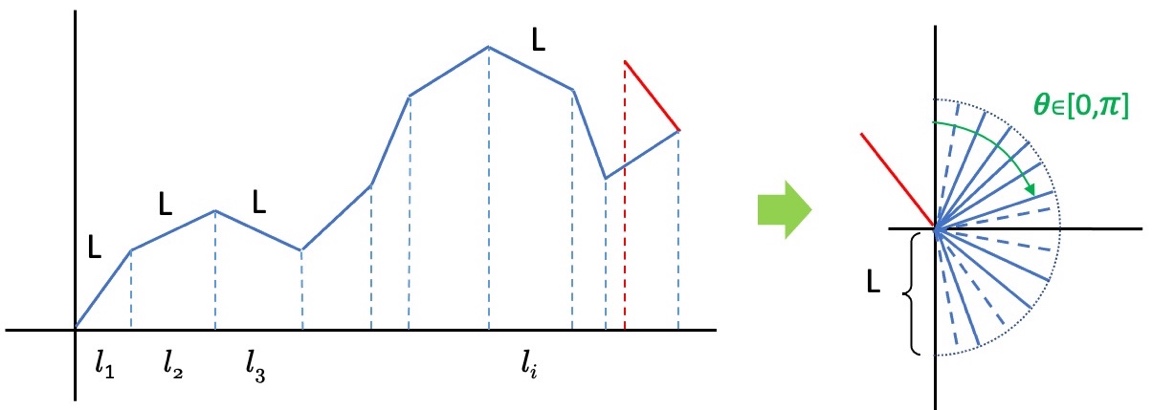


**Fig A. Unidimensional structure of joined segments of equal length *L* in 2D and spatial arrangement of the segments in a semicircle.**

The total length of the horizontally-projected unidimensional structure will be:

$$\sum_{i=1}^{N} l_{i}=\sum_{i=1}^{N} L \sin\left( \theta_{i} \right) \Longrightarrow\sum_{i=1}^{N} l_{i}=L \sum_{i=1}^{N} \sin\left( \theta_{i} \right).$$

Taking into account that the total length on the structure is *NL*:

$$\sum_{i=1}^{N} l_{i}=NL \frac{\sum_{i=1}^{N} \sin\left( \theta_{i} \right)}{N} =NL \frac{\sum_{i=1}^{N} \sin\left( \theta_{i} \right)}{\sum_{i=1}^{N} 1}.$$

Let us sort the segments in ascending order according to the value of their angles $\theta_{i}$ with the vertical positive axis (Fig. A right in S1 Text), so $\theta_{i-1}<\theta_{i} \forall i\in\left\{ 2, \ldots, N \right\}$, where $\theta_{1}=0$ and $\theta_{N}=\pi$. Thus, let us denote the difference between consecutive angles by $\Delta\theta_{i}\equiv\theta_{i}-\theta_{i-1}$. Therefore

$$\sum_{i=2}^{N} {\Delta\theta}_{i}=\left( \theta_{2}-\theta_{1} \right)+\left( \theta_{3}-\theta_{2} \right)+\left( \theta_{4}-\theta_{3} \right)+\ldots+\left( \theta_{N-2}-\theta_{N-3} \right)+\left( \theta_{N-1}-\theta_{N-2} \right)+\left( \theta_{N}-\theta_{N-1} \right)=$$

$$-\theta_{1}+\theta_{2}-\theta_{2}+\theta_{3}-\theta_{3}\ldots+\theta_{N-2}-\theta_{N-2}+\theta_{N-1}-\theta_{N-1}+\theta_{N}=\theta_{N}-\theta_{1}=\pi-0=\pi$$

independently of the number of segments *N*, so the sum of differences between consecutive angles does not diverge if $N\to\infty$.

Under this discrete approach, where there is a discrete set of possible angles $A\equiv\left\{ \alpha_{i} \right\} \mathrm{with} i\in\left\{ 1, \ldots, N \right\}$, the zero-anisotropy assumption implies that any segment of the unidimensional structure will be oriented with an angle $\theta_{i}$ belonging to the set A. When $N\to\infty$, which can be attained when the segment length $L\to0$ (i.e. when the axon-like structure is ‘broken’ into shorter and shorter segments). In this case, which represents the continuum limit, when the zero-anisotropy condition is fulfilled, the segment angles will be uniformly distributed in the interval $\left[ 0, \pi\right]$. In consequence, the difference between consecutive angles $\Delta\theta_{i}$ will verify that $\Delta\theta_{i}=\Delta\theta\to0$, or in other words: 1) the consecutive angles will be infinitely close to each other, and 2) we can assume that $\Delta\theta$ does not depend on *i* because of the angle uniform distribution.

Therefore, under the zero-anisotropy condition and considering $N\to\infty$ and $\Delta\theta\to0$, then by denoting *NL* by *L*_real_ and multiplying and dividing by $\Delta\theta$, it holds that:

$$l_{proj}\equiv\sum_{i=1}^{N} l_{i}=L_{real} \frac{\sum_{i=1}^{N} \sin\left( \theta_{i} \right)\Delta\theta}{\sum_{i=1}^{N} \Delta\theta}.$$

In the continuum limit:

$$\sum_{i=1}^{N} \sin\left( \theta_{i} \right)\Delta\theta\to\int_{0}^{\pi} \sin\left( \theta\right)d\theta=-\cos\left( \theta\right)|_{0}^{\pi}=2$$

$$\sum_{i=1}^{N} \Delta\theta\to\int_{0}^{\pi} d\theta=\theta|_{0}^{\pi}=\pi.$$

Therefore

$$l_{proj}=L_{real} \frac{\int_{0}^{\pi} \sin\left( \theta\right)d\theta}{\int_{0}^{\pi} d\theta}=L_{real} \frac{2}{\pi} ,$$

so

$$L_{real}=\frac{\pi}{2}l_{proj} .$$

It should be noted that the angle $\theta$ varies between 0 and $\pi$ because allowing different angles would introduce segments oriented ‘backward’, like the red segment in Fig A in S1 Text, so its projected segment would overlap with the projection of other segments and their lengths would be subtracted (close blue segment), which would lead to an inaccurate estimation of the real length. This assumption severely limits the application of the projection method to unidimensional structures embedded in 2D. Nonetheless, real axons are unidimensional structures in 3D, which will be the case addressed in the next section.

**Relationship between real and projected length of unidimensional structures in 3D 2D under zero-anisotropy condition**

Let us consider now a unidimensional structure of *M* segments of equal length *L* embedded in a three-dimensional space (Fig B left in S1 Text). For each segment $i\epsilon\left\{ 1,\ldots, M \right\}$ it holds:

$l_{i}=L \sin(\theta_{i})$,

where $l_{i}$ is the length of the projection of segment *i* in the horizontal plane (green segments in Fig B left in S1 Text). By arranging the segments with one of their extremes in the origin of coordinates, they will be enclosed in a sphere of radius *L* (Fig B middle in S1 Text), so they can be represented by their angles in the spherical coordinate system: $\theta_{i}$ as the angle between the segment and the positive vertical axis and $\varphi_{i}$ that determines the orientation of the projected segment in the horizontal (projection) plane (Fig B right in S1 Text).


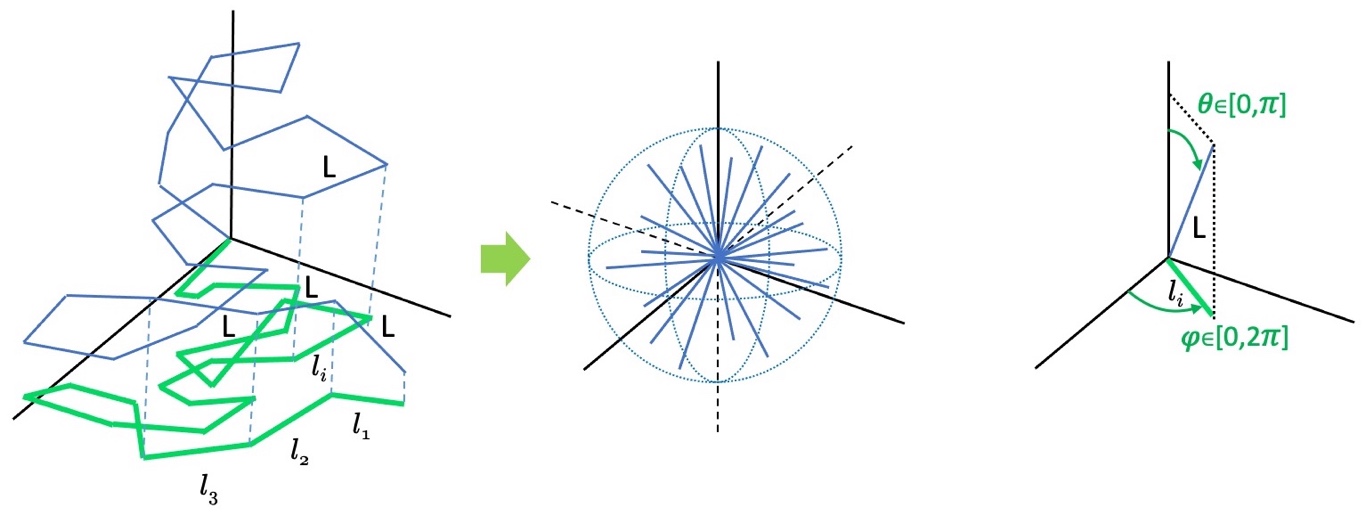


**Fig B. Unidimensional structure of joined segments of equal length *L* in 3D and spatial arrangement of the segments in a sphere of radius *L*.**

As in the previous section, the total length of the horizontally-projected unidimensional structure will be:

$$\sum_{i=1}^{M} l_{i}=\sum_{i=1}^{M} L \sin\left( \theta_{i} \right) \Longrightarrow\sum_{i=1}^{M} l_{i}=L \sum_{i=1}^{M} \sin\left( \theta_{i} \right)$$

Taking into account that the total length on the structure is *ML*:

$$\sum_{i=1}^{M} l_{i}=ML \frac{\sum_{i=1}^{M} \sin\left( \theta_{i} \right)}{M} =ML \frac{\sum_{i=1}^{M} \sin\left( \theta_{i} \right)}{\sum_{i=1}^{M} 1}$$

Following the same considerations and reasoning as in the previous section for the joint angles $\theta$ and $\varphi$, under zero-anisotropy condition when $M\to\infty\mathrm{and} \Delta\Omega\to0$, any value for the angles $\theta$ and $\varphi$ must be equally present ($\theta\in[0,\pi]$ and $\varphi\in[0,2\pi]$). Then, by considering spherical angles $\Omega$, the difference between its consecutive values will tend to zero, so $\Delta\Omega\to0$ (without loss of generality we can assume that $\Delta\Omega$ does not depend on *i*). Therefore, again by denoting *ML* by *L*_real_, it holds that:

$$l_{proj}\equiv\sum_{i=1}^{M} l_{i}=L_{real} \frac{\sum_{i=1}^{M} \sin\left( \theta_{i} \right)\Delta\Omega}{\sum_{i=1}^{N} \Delta\Omega}$$

In the continuum limit:

$$\sum_{i=1}^{M} \sin\left( \theta_{i} \right)\Delta\Omega\to\int_{0}^{2\pi} \int_{0}^{\pi} \sin\left( \theta\right)\sin\left( \theta\right)d\theta d\varphi=\int_{0}^{2\pi} d\varphi\int_{0}^{\pi} \sin^{2}\left( \theta\right)d\theta=2\pi\frac{\pi}{2}=\pi^{2}$$

$$\sum_{i=1}^{N} \Delta\Omega\to\int_{0}^{2\pi} \int_{0}^{\pi} \sin\left( \theta\right)d\theta d\varphi=\int_{0}^{2\pi} d\varphi\int_{0}^{\pi} \sin\left( \theta\right)d\theta=2\pi2=4\pi$$

$$=\pi$$

Therefore

$$l_{proj}=L_{real} \frac{\int_{0}^{2\pi} \int_{0}^{\pi} \sin\left( \theta\right)\sin\left( \theta\right)d\theta d\varphi}{\int_{0}^{2\pi} \int_{0}^{\pi} \sin\left( \theta\right)d\theta d\varphi}=L_{real} \frac{\pi^{2}}{4\pi}=L_{real} \frac{\pi}{4},$$

so

$$L_{real}=\frac{4}{\pi}l_{proj}$$

In this case, when the unidimensional structure is embedded in a 3D space, the limitation discussed in the previous 2D case does not exist, since the probability that the projections of two or more segments coincide is zero. Therefore, the above relationship between L_real_ and l_proj_ is fully applicable.
